# Lack of sex-specific differences in the associations between the dimensions of great vessels and exercise performance in amateur cyclists

**DOI:** 10.1371/journal.pone.0313165

**Published:** 2024-11-04

**Authors:** Michał J. Pytka, Remigiusz A. Domin, Mikołaj S. Żołyński, Jan Niziński, Tomasz Krauze, Barbara Więckowska, Andrzej Wykrętowicz, Przemysław Guzik

**Affiliations:** 1 Department of Cardiology – Intensive Therapy, Poznan University of Medical Sciences, Poznań, Poland; 2 University Centre for Sports and Medical Studies, Poznan University of Medical Sciences, Poznań, Poland; 3 Doctoral School, Poznan University of Medical Sciences, Poznan, Poland; 4 Department of Endocrinology, Metabolism and Internal Medicine, Poznan University of Medical Sciences, Poznań, Poland; 5 Department of Computer Science and Statistics, Poznan University of Medical Sciences, Poznań, Poland; Hamad Bin Khalifa University, QATAR

## Abstract

**Background:**

Endurance training enhances exercise capacity and triggers cardiovascular adaptations in both males and females. We investigated the relationship between the dimensions of great vessels and exercise capacity in amateur cyclists while considering sex differences.

**Methods:**

Using resting transthoracic echocardiography, we measured the dimensions of the main pulmonary artery (PA), aorta, and inferior vena cava (IVC) in 190 participants, who subsequently underwent a cardiopulmonary exercise test (CPET) until exhaustion.

**Results:**

The mean age of study participants was 30 years. Males (71%) exhibited a larger aortic annulus (approximately 3.5 mm, *p*<0.0001) and PA diameter (2.4 mm, *p*<0.0001) than females. No significant sex differences were found in expiratory or inspiratory IVC diameters. Males achieved greater peak exercise capacity, including workload, O_2_ consumption (VO_2_), and O_2_ pulse. Aortic and PA dimensions showed strong correlations with energy expenditure, workload, VO_2_, and O_2_ pulse. However, these correlations weakened when analyzed separately by sex. Multivariate linear regression revealed associations between CPET results, vessels size, and sex, with sex differences observed only in the intercepts—not in interactions between sex and vessels size. Despite males having better CPET results and larger vessels, the relationships between peak exercise capacity parameters and vessel dimensions were similar in both sexes.

**Conclusion:**

Larger vessel dimensions (of the aorta, PA, and IVC) were associated with greater peak exercise capacity in amateur cyclists, with no significant sex differences in these associations.

## Introduction

Various tissues and organs increase their demand for O_2_ and fuel during exercise [1, 2]. Energy production in the working muscles requires a constant supply of fuel and O_2_ and effective removal of heat and CO_2_. Simultaneously, various metabolites, including CO_2_, lactate, H^+^ ions, and adenosine, are produced during muscular exertion. These substances must be efficiently transported from the working muscles to other organs for processing. For example, CO_2_ is exhaled via the lungs, while lactate is utilized for gluconeogenesis in the liver or kidneys. Additionally, less active muscles can use lactate as a fuel source through the Cori cycle, which converts lactate into pyruvate for subsequent use in the Krebs cycle to produce ATP. The respiratory and cardiovascular systems adapt to match the demand for O_2_ delivery and increase the capacity to transport exercise metabolites. As a result, pulmonary ventilation, blood pumped by the heart and transported through the vessels increase [2–5].

Endurance exercise (including both training and physical work) causes the adaptation of many organs and systems to allow them to perform better. The heart of an athlete provides a classic example of such exercise-induced organ adaptation [1, 6]. The pulmonary and musculoskeletal systems also undergo exercise-induced remodeling, although the exact patterns of this process are not well understood [1, 7–9]. Many factors, including the type (endurance, power, mixed, and/or skill), volume, intensity, and duration of exercise; genetic predisposition; age; and sex, may contribute to such changes [6, 10].

An athlete’s heart is larger than that of a non-athlete, with thicker walls and dilated chambers, leading to increased myocardial mass. This translates into improved cardiac function, allowing highly trained individuals to achieve a greater cardiac output with a lower heart rate (HR) [1]. During exercise, cardiac output can increase up to 8-fold, reaching 40 L/min, compared to the resting 5 L/min [1]. Blood flow to muscles increases significantly (up to a 30-fold increase in elite athletes), while e.g. the digestive system receives less perfusion [11]. Exercise also benefits the vascular system by reducing arterial wall thickness and increasing lumen diameter, leading to decreased vascular resistance in muscle arteries during exercise [1].

Both males and females experience the effects of endurance exercise, which lead to adaptation of cells, tissues and organs. However, there are variations due to differences in body size and composition; sex hormones; genetics; and environmental factors like diet, lifestyle, training time, pregnancies, and stress [12]. Females generally have smaller cardiac chambers and mass and smaller aortic diameters than men. These anatomical differences might influence how the heart responds to exercise in males and females [12, 13], however research in this topic is lacking.

Despite extensive research on the cardiovascular system, a crucial gap remains in our understanding of how the dimensions of great vessels relate to exercise capacity in males and females This study examined a group of amateur cyclists with varying exercise levels to assess the relationship between dimensions of the aorta, main pulmonary artery (PA), and inferior vena cava (IVC) with exercise capacity. We further investigated whether these associations existed separately in males and females and if these associations differed by sex. This research is exploratory, and no hypothesis regarding sex differences was formulated. Consequently, we aim to establish associations rather than causality.

## Materials and methods

### Bioethical issues

This paper presents results of a project that was approved by the Bioethics Committee of Poznan University of Medical Sciences (decision 693/20). The study was conducted according to the Declaration of Helsinki [14]. All data were treated confidentially and anonymized for storage and analysis. The data were collected, stored, and analyzed in the REDCap data capture tools hosted at Poznan University of Medical Sciences [15].

### Study group recruitment

We enrolled 215 consecutive adult volunteers who claimed to be recreationally physically active and cycled regularly for a cross-sectional, observational study with a single timepoint assessment. The recruitment took place between December 1^st^ 2020, and April 30^th^ 2023. Details of all participants have already been published [16]. The enrolment included consecutive volunteers who responded to the recruitment call who wanted to participate in a study to evaluate their cardiovascular function by electrocardiogram (ECG), echocardiography (ECHO), and CPET. All participants took part voluntarily and were informed about the study and the fact that they could abandon the study at any time. Written informed consent was collected from all volunteers.

For this substudy, we selected participants with complete data from CPET and ECHO measurements concerning great vessel dimensions. Therefore, 190 subjects were analyzed. The inclusion criteria were being recreationally physically active and cycling regularly for at least 1 h per week to ensure the participation of a wide variety of amateur cyclists. Individuals with chronic diseases or who were taking medications were excluded from the study. Allowed substances included vitamin D, dietary supplements, and oral contraceptives for women.

### Health status

Physicians performed a comprehensive pre-participation screening, including a detailed medical and family history, physical examination, and blood pressure measurement. A resting 12-lead ECG was performed to detect abnormalities such as arrhythmias, conduction problems, signs of left ventricle (LV) or right ventricle (RV) hypertrophy, pre-excitation syndromes, or channelopathies. In addition, resting transthoracic ECHO assessed cardiac structure and function to exclude clinically significant abnormalities such as moderate to severe valve stenosis or regurgitation (e.g., mitral regurgitation or aortic stenosis). Six individuals were excluded due to LV hypertrophy or moderate mitral or tricuspid regurgitation.

### Anthropometric measurements and physical activity assessment

Body height and weight were measured to calculate body mass index (BMI). To assess physical activity, we used the International Physical Activity Questionnaire (IPAQ) [17], as well as additional questions regarding basic characteristics of training (cycling sessions per week and hours of cycling training per session and per week). MET-min (metabolic equivalent of task–minutes) were calculated according to IPAQ interpretation guidelines [17]. The MET-min is a parameter showing the volume of exercise undertaken by a participant.

### Spirometry

Participants underwent baseline resting spirometry (Vyntus CPX, Vyaire Medical, IL, USA) after resting in a sitting position for at least 5 min. The measured values included forced expiratory volume in 1 s (FEV1). Maximal voluntary ventilation was calculated using the formula FEV1 × 40 and defined the limit of breathing reserve for CPET [18]. The test was performed until five results met the criteria for adequate quality [18, 19], and the mean results from the three best repetitions were used for analysis.

### Cardiopulmonary exercise test

CPET was performed on a cycle ergometer (Excalibur Sport 2, Lode, Groningen, The Netherlands) using a CPET system (Vyntus CPX, Vyaire Medical, IL, USA). HR was recorded using a chest-strap HR monitor (Polar H10, Polar, Kempele, Finland). The tidal volume and content of O_2_ and CO_2_ in the inhaled and exhaled air were measured using the breath-by-breath method.

The CPET was executed according to the Association for Respiratory Technology & Physiology guidelines [18] using an individualized incremental ramp protocol tailored to the participants’ current physical performance. Additionally, we took into consideration the IPAQ result–MET-min and IPAQ category. For participants in the lower physical activity category we aimed for the peak load after 10 minutes of exercise to reach 2.5–3.5 W/kg, for the moderate physical activity category– 3.5–4.5 W/kg and for the high physical activity category– 4.5–5.5 W/kg. The resting phase lasted 2 minutes, the warmup phase (pedaling with no additional load) lasted 2 minutes. The main phase of the test (incremental ramp protocol) aimed to last 8−12 min [18, 20] and was terminated at the will of the participant at peak effort. The participants were encouraged to exert maximal effort; they could increase cadence, muscle strain, or both. During peak exercise, all participants reached at least 8 points in the modified Borg scale, the median RER was 1.24 (RER > 1.15 defines high effort), and all reached >90% of predicted maximal heart rate and/or maximal VO_2_. During the recovery phase, monitoring continued for at least 10 min while the participant was seated.

The recorded parameters, which were used for further analysis, included:

- HR: heart rate;
- VO_2_: the volume of O_2_ consumed per min;
- O_2_ pulse: the ratio of VO_2_ to HR;
- VCO_2_: the volume of CO_2_ produced per min;
- VE: minute ventilation;
- RER: respiratory exchange ratio; and
- EE: energy expenditure.

EE, expressed as kcal per min, was calculated using the Weir formula for indirect calorimetry [21]:

$$EE=\left(3.9*{VO}_{2}+1.1*{VCO}_{2}\right)\;\left[\frac{kcal}{min}\right].$$

The recorded time points included averaged breath-by-breath values from the last 15 s of the rest and peak exercise phases, the latter of which usually occurred before the end of the cycling.

### Echocardiography

Echocardiography was performed in all participants according to the American Society of Echocardiography guidelines [22]. Experienced cardiologists used a 3.5-MHz transducer on a Vivid E95 or E9 ECHO machine from General Electric Healthcare Technologies (Chicago, IL, USA). The IVC was measured in the subcostal view approximately 1 cm before the entrance to the right atrium. The IVC expiratory and inspiratory diameters were obtained using the M-mode while the patient was breathing deeply. In the parasternal long axis view, the following diameters of the aorta were measured: aortic annulus diameter, aortic sinus of Valsalva diameter, and aortic sinotubular junction (STJ) diameter. From the suprasternal view, we quantified the diameter of the arch of the aorta. The diameter of the abdominal aorta was measured in the longitudinal plane, in the subcostal view focused on the proximal abdominal aorta. Finally, in the parasternal short axis view, focused on the PA, the diameters of the PA and of the left pulmonary artery (LPA) and right pulmonary artery (RPA) (1 cm after their takeoff) were measured. Most measurements of arteries were taken using the leading-to-leading method. The aortic annulus and IVC dimensions were measured using the trailing-to-leading approach.

The echocardiographic data (images and cine loops) were analyzed using TOMTEC Imaging Systems (TOMTEC-ARENA Build No. 544347, TOMTEC Imaging Systems GmbH, Unterschleissheim, Germany, distributed by Phillips, Amsterdam, The Netherlands).

**Fig 1:**
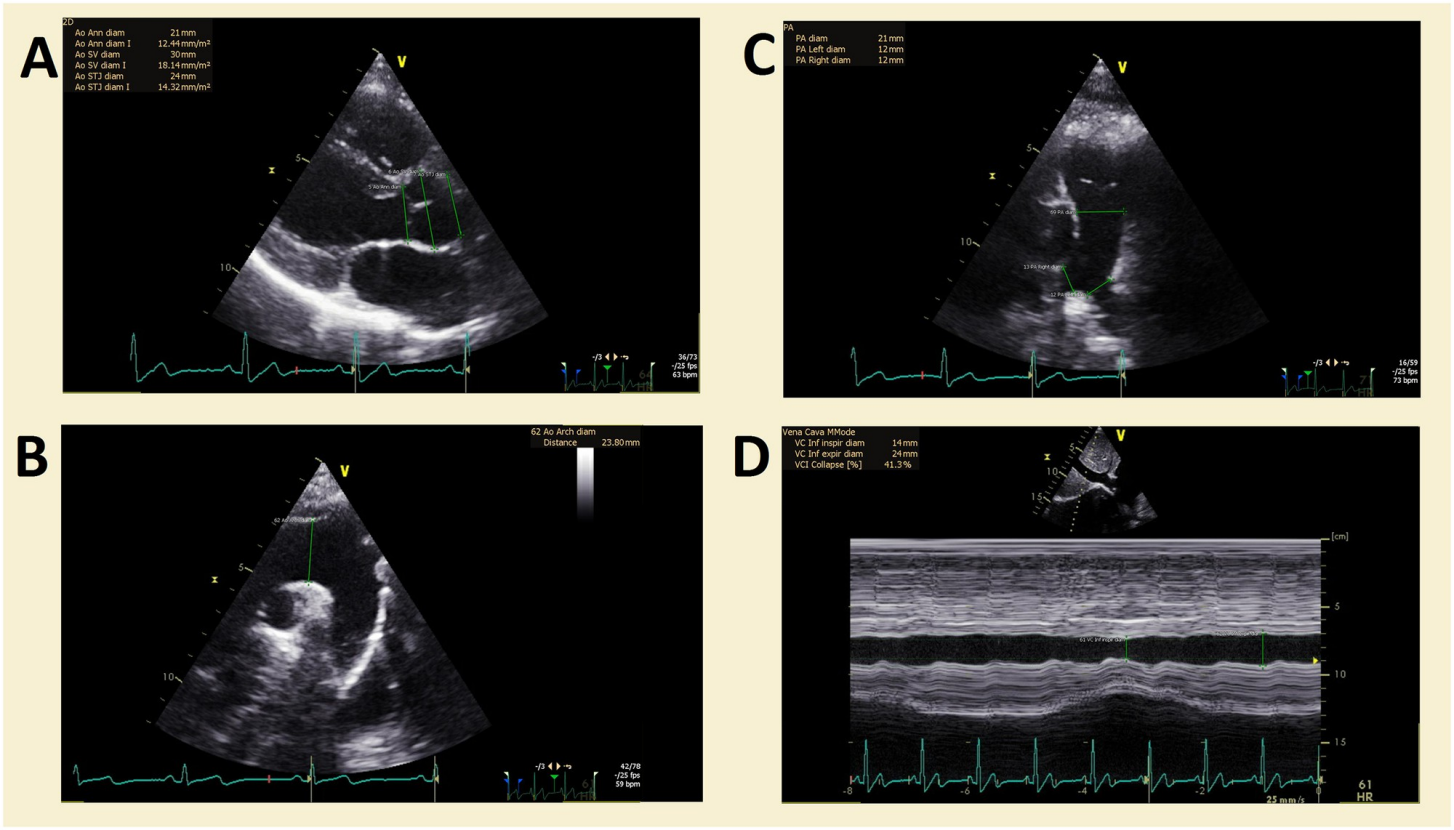
An example of echocardiographic measurements for the study. A–measurements of the aorta in the parasternal long axis (aortic annulus, sinus of Valsalva and sinotubular junction diameters). B–measurement of the arch of the aorta in the suprasternal view. C–measurement of the diameter of the main pulmonary artery and the left and right pulmonary arteries in the modified parasternal short axis with focus on the main pulmonary artery. D–measurement of the inferior vena cava during inspiration and expiration–measured in M-mode.

An example of echocardiographic measurements is presented in Fig 1.

### Statistical analysis

QQ-plots and the D’Agostino–Pearson test were used to test the normality of the data distributions [23]. The mean ± standard deviation (SD) summarizes data with a normal distribution, while the median and difference between the 75^th^ and 25^th^ percentiles (interquartile range) describe data that are not normally distributed. Depending on the data distribution, comparisons between the sexes were made using either the parametric Student’s *t*-test or the non-parametric Mann–Whitney test. Associations between the dimensions of great vessels and peak exercise performance parameters from CPET, as well as the influence of sex, were preliminarily examined using Pearson correlation (all these parameters were normally distributed). Multivariate linear regression models analyzed the effects of vascular dimensions (*x*) and sex and the interactions of these parameters (*x* × sex) on exercise capacity parameters (HR, VO_2_, VO_2_·kg^−1^, VCO_2_, VE, load, O_2_ pulse, and RER). Within these models, the estimate for *x* represents the direct association between a peak CPET parameter and a specific great vessel dimension. The sex estimate (0 = female, 1 = male) indicates the presence of sex differences in peak CPET parameters; a significant estimate indicates significant sex differences in the dependence of CPET on vessel dimensions. The interaction term *x* × sex reflects whether the association between specific vessel dimensions and peak CPET parameters differs between male and female cyclists. This approach essentially compares two separate regressions for each sex: the sex effect compares the intercepts, while the *x* × sex interaction compares the slopes of these regressions. As our primary aim was to determine whether the slopes of the relationships between exercise capacity and the dimensions of great vessels differed between males and females, we focused on reporting only the *p*-value for the interaction term between vessels size and sex. In all analyses, only *p*-values <0.05 were considered significant. JMP^®^ Pro 17.0.0 (622753) (JMP Statistical Software, Cary, NC, USA) was used for statistical analyses.

## Results

### Study group characteristics

Table 1 summarizes the characteristics of the 190 participants (134 males, 71%; 56 females, 29%). Males were taller (by 13 cm) and heavier (by 19.2 kg) than females, with a BMI 3.1 kg·m^−2^ higher. However, median age and measures of exercise volume (e.g., hours per week and intensity) were similar between the sexes. The mean value of MET minutes per week was 4225. The IPAQ classified most participants (56%) as very active, 34% moderately active, and 10% less active. Cycling was the main sport activity for all participants, and the median number of cycling sessions per week was 4. The median cycling training time was 3.5 hours/week, with a minimum of 2 hours per week (only 22 participants, 11%). Other sports activities undertaken in the study group included running (40%), gym (25%), swimming (19%), team sports (9%), and others (31%).

**Table 1:** Clinical characteristics of studied male and female amateur cyclists and their peak cardio-pulmonary exercise test results.

Parameter		Males (N = 134)		Females (N = 56)		p value
		Median	IQR	Median	IQR	
Height [cm]		181	178–185	168	165–172	<0.0001*
Weight [kg]		79.7	72.4–87.8	60.0	55.0–66.3	<0.0001*
BMI [kg·m^-2^]		24.5	22.7–26.5	21.4	20.0–23.2	<0.0001*
Age [years]		29	25–37	26	22–35	0.1432
Exercise days per week [days]		4	3–5	4	3–5	0.5470
Weekly training time [hours/week]		6	4–9	6.5	4–8	0.5653
Weekly cycling time [hours/week]		4	2.5–6.0	3	2.5–5.1	0.1566
MET-min [per week]		3470	1479–6124	2949	1424–5295	0.7607
CPET duration [min]		10.6	9.7–11.5	10.0	9.0–11.1	0.2136
		Mean ± SD		Mean ± SD		
Resting ECHO	Ao Annulus [mm]	22.6 ± 2.3		19.1 ± 1.9		<0.0001*
	Ao SV [mm]	32.9 ± 3.2		28.5 ± 2.7		<0.0001*
	Ao STJ [mm]	26.8 ± 3.2		24.1 ± 2.7		<0.0001*
	Ao Arch [mm]	24.5 ±2.7		22.1 ± 2.4		<0.0001*
	Ao Abd [mm]	17.9 ± 1.8		16.1 ± 1.6		<0.0001*
	PA [mm]	21.4 ± 2.8		19.0 ± 2.16		<0.0001*
	LPA [mm]	16.2 ± 2.1		14.4 ± 1.8		<0.0001*
	RPA [mm]	15.9 ± 2.1		14.2 ± 1.7		<0.0001*
	IVC inspiration [mm]	10.2 ± 3.8		10.6 ± 3.2		0.5718
	IVC expiration [mm]	20.7 ± 5.8		21.0 ± 4.4		0.7379
Rest before exercise	HR [beats·min^-1^]	82.9 ± 15.1		81.38 ± 15.4		0.5339
	O_2_pulse [mL·beat^-1^]	6.2 ±2.		4.3 ± 1.3		<0.0001*
	RER	0.90 ± 0.14		0.89 ± 0.13		0.5258
	VCO_2_ [L·min^-1^]	0.44 ± 0.17		0.30 ± 0.09		<0.0001*
	VO_2_ [L·min^-1^]	0.50 ± 0.17		0.35 ± 0.09		<0.0001*
	VO_2_kg [mL·min^-1^·kg^-1^]	5.70 ± 2.83		4.50 ± 2.30		0.0052*
	VE [L·min^-1^]	16.4 ± 5.1		12.5 ± 3.4		<0.0001*
	EE [kcal·min^-1^]	2.45 ± 1.44		1.66 ± 0.66		<0.0001*
Peak exercise	HR [beats·min^-1^]	184.7 ± 11.0		182.1 ± 11.2		0.1359
	Load [W]	342.7 ± 63.73		229.1 ± 45.9		<0.0001*
	Load per body mass [W·kg^-1^]	4.31 ± 0.93		3.72 ± 0.64		<0.0001*
	O_2_pulse [mL·beat^-1^]	20.7 ± 3.5		14.0 ± 2.8		<0.0001*
	RER	1.24 ± 0.07		1.22 ± 0.08		0.0209*
	VCO_2_ [L·min^-1^]	4.72 ± 0.74		3.08 ± 0.54		<0.0001*
	VO_2_ [L·min^-1^]	3.81 ± 0.60		2.54 ± 0.47		<0.0001*
	VO_2_·kg^-1^ [mL·min^-1^·kg^-1^]	43.3 ± 12.9		33.5 ± 13.4		<0.0001*
	VE [L·min^-1^]	159.7 ± 30.8		106.1 ± 19.0		<0.0001*
	EE [kcal·min^-1^]	20.17 ± 4.52		13.38 ± 3.49		<0.0001*

Comparisons between males and females were made using the parametric student’s t-test (for normally distributed data) or the non-parametric Mann-Whitney test (for not normally distributed data).

Abbreviations: Ao Abd–abdominal aorta diameter; Ao Annulus–aortic annulus diameter; Ao Arch–aortic arch diameter; Ao STJ–aortic sinotubular junction diameter; Ao SV–aortic sinus Valsalva diameter; BMI–body mass index; CPET- cardio-pulmonary exercise test; EE–energy expenditure; HR–heart rate; IVC–inferior vena cava diameter; LPA–left pulmonary artery diameter; LVOT–left ventricle outflow tract diameter; MET–the metabolic equivalent of task; O_2_ pulse—the ratio of VO_2_ to HR; PA–main pulmonary artery diameter; RER–respiratory exchange ratio; RPA–right pulmonary artery diameter; SD–standard deviation. VCO_2_ –the volume of produced CO_2_; VE–minute ventilation; VO_2_ –the volume of consumed O_2_; VO_2_·kg^-1^ –the volume of consumed O_2_ per kilogram of body weight;

### Echocardiography

Table 1 summarizes the echocardiographic measurements of the aorta, PA, and IVC. Males had larger aortic diameters than females at the annulus, sinus of Valsalva, STJ, aortic arch, and proximal abdominal aorta. The diameter of the PA was 2.4 mm larger in males, and the diameters of the LPA and RPA were 1.8 and 1.7 mm larger, respectively. The diameter of the IVC was 20.8 mm during expiration and 10.3 mm during inspiration and was similar in males and females. The absolute and relative increases in IVC diameter during expiration were similar in males and females (10.5 vs. 10.6 mm and 51.1% vs. 50.6%, respectively).

### Cardiopulmonary exercise test

The data from CPET at rest and during peak exercise are presented in Table 1. During peak exercise, the HR increased by 225%, from 82 to 184 bpm, and was comparable between males and females. Males achieved higher peak load (343 vs. 229 W), as well as load per unit body mass (4.31 vs. 3.72 W·kg^−1^). For all participants, breathing frequency increased by 325%, reaching 51 breaths per min at peak exercise, and the peak VE was 144 L·min^−1^. O_2_ pulse was higher in males and reached 20.7 mL·beat^−1^ (334% increase) compared to 14.0 mL·beat^−1^ (326% increase) in females. The peak RER was 1.24, indicating very high effort by the participants. The data are available in the supporting information–S1 Table.

### Associations between the dimensions of great vessels and exercise capacity

**Fig 2:**
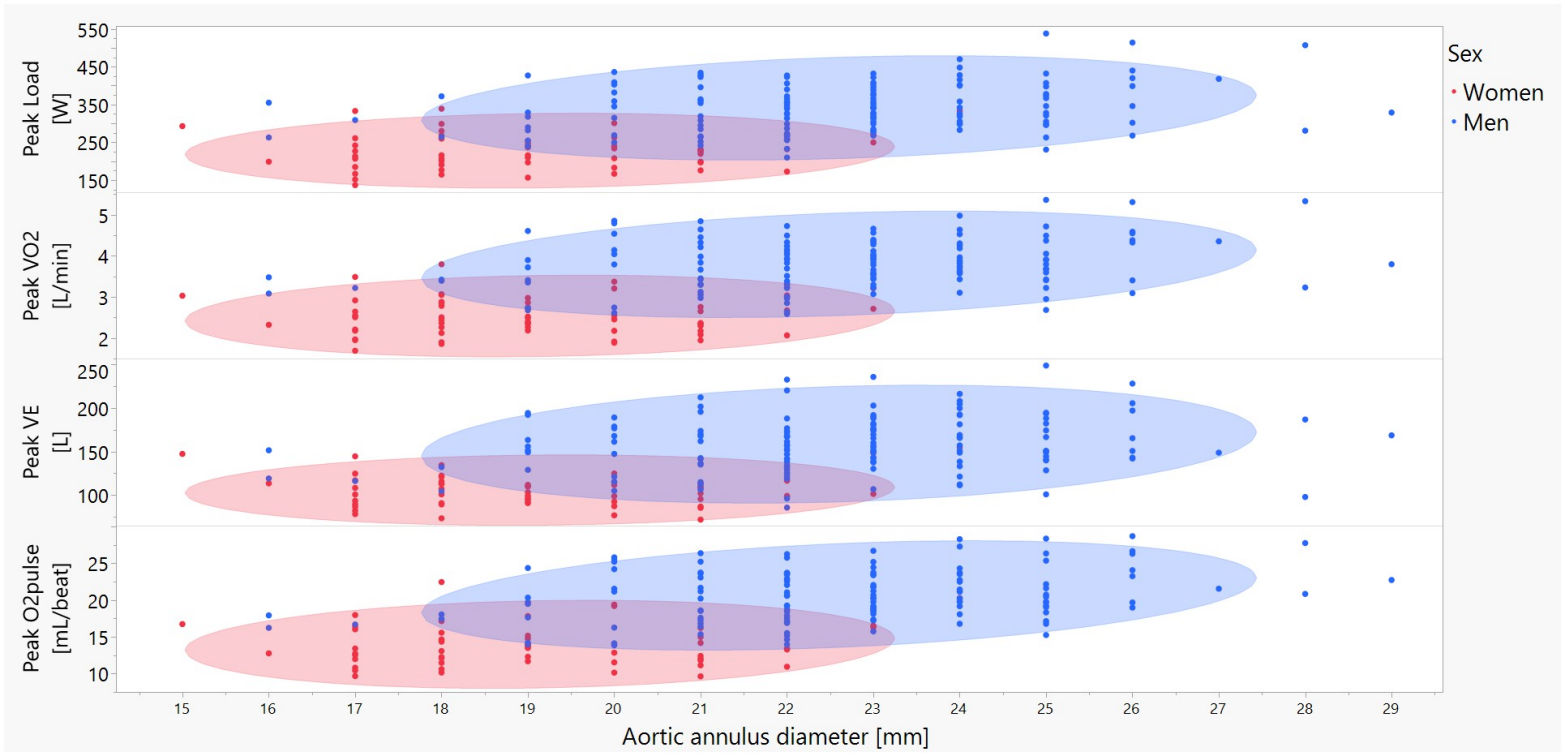
Correlations between aortic annulus diameter and peak exercise capacity parameters from CPET. The red dots and shaded ellipses represent females, the blue dots and shades represent males. Abbreviations: O_2_ pulse—the ratio of VO_2_ to HR; VE–minute ventilation; VO_2_ –the volume of consumed O_2_.

The diameter of the aorta, particularly at the levels of the annulus, the sinuses of Valsalva, and the proximal abdominal segment below the diaphragm, as well as of the LPA, showed significant positive correlations of moderate strength with peak EE, workload, O_2_ pulse, VCO_2_, VO_2_, and VE. Interestingly, peak HR showed a weak negative correlation with the same dimensions. The IVC showed weak or nonsignificant correlations with the same exercise capacity parameters, with the exception of O_2_ pulse. All correlations were generally weaker when analyzed separately for males and females. Fig 2 illustrates some of the strongest correlations observed, focusing on the aortic annulus and selected peak CPET parameters.

In Table 2, we present the results of the Pearson correlation coefficient analysis for associations between the dimensions of great vessels and exercise capacity. Generally, stronger correlations were found in the whole study group, and weaker correlations were found in the subgroups for each sex. The strongest recorded correlations were moderate (*r*≥0.4) and were found between dimensions of the aorta and of the PA and peak EE, load, O_2_ pulse, VCO_2_, and VO_2_, as well as between dimensions of the aorta and VE.

**Table 2:** Pearson correlation coefficient for associations between the dimensions of great vessels and exercise capacity parameters in the study group and in sex-subgroups.

y	x	Study group (N = 190)		Females (N = 56)		Males (N = 134)	
		r	p	r	p	r	p
Peak EE	Ao Annulus	0.56	<0.0001	0.11	0.4136	0.27	0.0019
	Ao SV	0.50	<0.0001	0.25	0.0688	0.17	0.0474
	Ao STJ	0.33	<0.0001	0.12	0.3634	0.09	0.3147
	Ao Arch	0.36	<0.0001	0.25	0.0629	0.09	0.2964
	Ao Abd	0.49	<0.0001	0.39	0.0034	0.29	0.0008
	PA	0.44	<0.0001	0.28	0.0393	0.23	0.0075
	LPA	0.46	<0.0001	0.33	0.0135	0.29	0.0006
	RPA	0.42	<0.0001	0.30	0.0236	0.23	0.0085
	IVC inspiration	0.12	0.1128	0.20	0.1324	0.21	0.0131
	IVC expiration	0.16	0.0267	0.27	0.0441	0.26	0.0027
Peak HR	Ao Annulus	-0.07	0.3452	-0.03	0.8279	-0.21	0.0132
	Ao SV	-0.25	0.0006	-0.32	0.0172	-0.39	<0.0001
	Ao STJ	-0.27	0.0002	-0.29	0.0317	-0.35	<0.0001
	Ao Arch	-0.23	0.0013	-0.12	0.3717	-0.36	<0.0001
	Ao Abd	-0.19	0.0074	-0.27	0.0469	-0.26	0.0021
	PA	-0.17	0.0159	-0.17	0.2192	-0.26	0.0022
	LPA	-0.33	<0.0001	-0.47	0.0003	-0.38	<0.0001
	RPA	-0.31	<0.0001	-0.42	0.0013	-0.37	<0.0001
	IVC inspiration	-0.11	0.1413	-0.04	0.7776	-0.13	0.1446
	IVC expiration	-0.21	0.0039	-0.27	0.0451	-0.19	0.0284
Peak Load	Ao Annulus	0.52	<0.0001	0.11	0.4214	0.24	0.0047
	Ao SV	0.48	<0.0001	0.25	0.0607	0.18	0.0328
	Ao STJ	0.33	<0.0001	0.16	0.2258	0.10	0.2292
	Ao Arch	0.35	<0.0001	0.25	0.0646	0.11	0.1869
	Ao Abd	0.46	<0.0001	0.36	0.0065	0.26	0.0023
	PA	0.41	<0.0001	0.26	0.052	0.21	0.0130
	LPA	0.44	<0.0001	0.25	0.0623	0.28	0.0011
	RPA	0.40	<0.0001	0.24	0.0739	0.22	0.0113
	IVC inspiration	0.13	0.0715	0.17	0.2105	0.22	0.0099
	IVC expiration	0.18	0.0152	0.23	0.0822	0.26	0.0023
Peak O_2_pulse	Ao Annulus	0.56	<0.0001	0.14	0.3197	0.32	0.0002
	Ao SV	0.56	<0.0001	0.33	0.0122	0.31	0.0003
	Ao STJ	0.40	<0.0001	0.21	0.1228	0.23	0.0089
	Ao Arch	0.43	<0.0001	0.29	0.033	0.24	0.0062
	Ao Abd	0.55	<0.0001	0.48	0.0002	0.38	<0.0001
	PA	0.47	<0.0001	0.32	0.0166	0.31	0.0003
	LPA	0.55	<0.0001	0.48	0.0002	0.43	<0.0001
	RPA	0.51	<0.0001	0.45	0.0005	0.36	<0.0001
	IVC inspiration	0.15	0.0437	0.20	0.1480	0.25	0.0036
	IVC expiration	0.22	0.0022	0.35	0.0087	0.32	0.0002
Peak RER	Ao Annulus	-0.01	0.8769	-0.19	0.1688	-0.12	0.1594
	Ao SV	0.00	0.9989	-0.11	0.4005	-0.11	0.1989
	Ao STJ	-0.11	0.1216	-0.14	0.3095	-0.22	0.0115
	Ao Arch	-0.10	0.1910	-0.12	0.3847	-0.20	0.0189
	Ao Abd	-0.18	0.0131	-0.41	0.0019	-0.23	0.0082
	PA	0.03	0.6604	0.08	0.5781	-0.08	0.3489
	LPA	-0.16	0.0241	-0.23	0.0906	-0.26	0.0023
	RPA	-0.16	0.0265	-0.28	0.0400	-0.24	0.0059
	IVC inspiration	0.01	0.8644	0.08	0.5543	0.00	0.9556
	IVC expiration	-0.08	0.2887	-0.12	0.3622	-0.06	0.5079
Peak VCO_2_	Ao Annulus	0.55	<0.0001	0.06	0.6440	0.24	0.0045
	Ao SV	0.49	<0.0001	0.21	0.1216	0.14	0.1125
	Ao STJ	0.30	<0.0001	0.09	0.5246	0.04	0.6728
	Ao Arch	0.33	<0.0001	0.22	0.1076	0.04	0.6418
	Ao Abd	0.45	<0.0001	0.28	0.0363	0.23	0.0084
	PA	0.43	<0.0001	0.29	0.0311	0.22	0.0116
	LPA	0.42	<0.0001	0.26	0.0501	0.23	0.0079
	RPA	0.38	<0.0001	0.22	0.0962	0.17	0.0499
	IVC inspiration	0.11	0.1295	0.22	0.1058	0.21	0.0166
	IVC expiration	0.14	0.0499	0.23	0.0817	0.24	0.0055
Peak VE	Ao Annulus	0.50	<0.0001	0.08	0.5408	0.21	0.0161
	Ao SV	0.46	<0.0001	0.26	0.0570	0.14	0.0961
	Ao STJ	0.25	0.0006	0.10	0.4497	-0.02	0.8352
	Ao Arch	0.32	<0.0001	0.14	0.2958	0.10	0.2589
	Ao Abd	0.39	<0.0001	0.28	0.0397	0.14	0.1053
	PA	0.36	<0.0001	0.29	0.0310	0.12	0.1503
	LPA	0.36	<0.0001	0.33	0.0136	0.13	0.142
	RPA	0.34	<0.0001	0.30	0.0264	0.11	0.1941
	IVC inspiration	0.05	0.4695	0.20	0.1353	0.09	0.3145
	IVC expiration	0.11	0.1252	0.27	0.0435	0.15	0.0800
Peak VO_2_	Ao Annulus	0.55	<0.0001	0.12	0.3621	0.27	0.0018
	Ao SV	0.50	<0.0001	0.25	0.0619	0.18	0.0351
	Ao STJ	0.34	<0.0001	0.13	0.3393	0.11	0.2170
	Ao Arch	0.37	<0.0001	0.26	0.0579	0.12	0.1815
	Ao Abd	0.50	<0.0001	0.41	0.0016	0.30	0.0004
	PA	0.44	<0.0001	0.27	0.0405	0.23	0.0065
	LPA	0.48	<0.0001	0.35	0.0085	0.32	0.0002
	RPA	0.43	<0.0001	0.33	0.0136	0.24	0.0045
	IVC inspiration	0.12	0.1034	0.20	0.1469	0.22	0.0119
	IVC expiration	0.17	0.0195	0.28	0.0352	0.26	0.002
Peak VO_2_·kg^-1^	Ao Annulus	0.07	0.3170	-0.32	0.0178	-0.10	0.2722
	Ao SV	0.20	0.0055	0.03	0.8110	0.03	0.7222
	Ao STJ	0.00	0.9638	-0.17	0.2176	-0.12	0.1613
	Ao Arch	0.00	0.9464	-0.08	0.5704	-0.17	0.0452
	Ao Abd	0.22	0.0022	-0.02	0.896	0.14	0.0961
	PA	0.26	0.0004	0.04	0.7861	0.18	0.0328
	LPA	0.35	<0.0001	0.26	0.0509	0.26	0.0022
	RPA	0.29	<0.0001	0.28	0.0355	0.17	0.0482
	IVC inspiration	0.16	0.0288	0.10	0.4862	0.21	0.0129
	IVC expiration	0.18	0.0116	0.11	0.406	0.23	0.0068

The Pearson correlation coefficient explored associations between peak exercise performance parameters from CPET and the dimensions of the great vessels from ECHO.

Abbreviations: Ao Abd–abdominal aorta diameter; Ao Annulus–annulus of the aorta diameter; Ao Arch—aortic arch diameter; Ao STJ—aortic sinotubular junction diameter; Ao SV—aortic sinus of Valsalva diameter; EE–energy expenditure; IVC expiration–inferior vena cava diameter during expiration; IVC inspiration–inferior vena cava diameter during inspiration; HR–heart rate; LPA–left pulmonary artery diameter; O_2_pulse—the ratio of VO_2_ to HR; PA–main pulmonary artery diameter; RER–respiratory exchange ratio; RPA–right pulmonary artery diameter; VCO_2_ –the volume of produced CO_2_; VE–minute ventilation; VO_2_ –the volume of consumed O_2_; VO_2_·kg^-1^ –the volume of consumed O_2_ per kilogram of body weight

In the whole study group, larger aortic dimensions were correlated with higher peak EE, load, O_2_ pulse, VCO_2_, VO_2_, and VO_2_·kg^−1^ and with lower peak HR. Specifically, the strongest correlations included aortic annulus diameter with: EE (*r* = 0.56, *p*<0.0001), O_2_ pulse (*r* = 0.56, *p*<0.0001), VCO_2_ (*r* = 0.55, *p*<0.0001), and VO_2_ (*r* = 0.55, *p*<0.0001); aortic sinus of Valsalva diameter with O_2_ pulse (*r* = 0.56, *p*<0.0001); proximal abdominal aorta diameter with O_2_ pulse (*r* = 0.55, *p*<0.0001); and LPA with O_2_pulse (*r* = 0.55, *p*<0.0001). Larger dimensions of the thoracic aorta were correlated with higher O_2_ pulse and lower HR in males. Proximal abdominal aorta diameter was larger in cyclists (both males and females) who achieved higher peak EE, load, O_2_ pulse, VO_2_ and RER. The abdominal aorta was additionally correlated with higher VCO_2_ in males and with higher VE in females.

Larger dimensions of the PA and its branches were correlated with higher EE, load, O_2_ pulse, VCO_2_, VE, VO_2_, and VO_2_·kg^−1^ and with lower HR in all cyclists. For males, the correlations were significant between the diameters of the PA, LPA, and RPA and EE, HR, O_2_ pulse, VCO_2_, VO_2_, and VO_2_·kg^−1^, as well as between the diameters of the RPA and LPA and RER. In females, fewer correlations were found: between PA diameter and EE, O_2_ pulse, VCO_2_, VE, VO_2_; LPA diameter and EE, VO_2_, O_2_ pulse and VE; RPA diameter and EE, HR, O_2_ pulse, RER, VO_2_, VO_2_·kg^−1^ and VE.

The IVC expiratory and inspiratory diameters were correlated with higher EE, load, O_2_ pulse, VCO_2_, and VO_2_·kg^−1^ in males. In females, only the IVC expiratory diameter was associated with higher EE, O_2_ pulse, VE, and VO_2_ and lower HR.

### Sex-specific associations between peak CPET parameters and resting dimensions of great vessels

Tables 3 and 4 summarize the potential influence of the dimensions of great vessels, sex, and their interaction on peak CPET parameters. The estimate for each vessel dimension (*x*) represents its direct effect on peak CPET. The estimate for sex (0 = female, 1 = male) indicates sex differences in peak CPET. Significant interaction terms (*x* × sex) indicate that the relationship between a vessel dimension and peak CPET differs between males and females. However, no interactions were statistically significant, indicating that these relationships were comparable in both sexes. Therefore, the final regressions (Table 4) included only vessel dimension (*x*) and sex as independent variables, without interaction terms.

**Table 3:** Table 3 shows the p-values for the interaction terms between resting large vessel dimensions and sex in their influence on peak CPET parameters. As none of these interaction terms were statistically significant and influenced the linear regression models, the estimates for vessel dimensions and sex are omitted from this table for clarity. The final regression models without interaction terms are shown in Table 4. Data presented for N = 190 (134 males, 56 females).

y	x	p-value for the x and sex interaction	x	p-value for the x and sex interaction
Peak EE	Ao Annulus	0.3182	PA	0.7773
	Ao SV	0.7553	LPA	0.9442
	Ao STJ	0.8840	RPA	0.7152
	Ao Arch	0.4285	IVC inspiration	0.8746
	Ao Abd	0.7394	IVC expiration	0.9276
Peak HR	Ao Annulus	0.3213	PA	0.8434
	Ao SV	0.9835	LPA	0.2535
	Ao STJ	0.9575	RPA	0.3761
	Ao Arch	0.2021	IVC inspiration	0.6633
	Ao Abd	0.7893	IVC expiration	0.3849
Peak load	Ao Annulus	0.3600	PA	0.8501
	Ao SV	0.8528	LPA	0.7116
	Ao STJ	0.8358	RPA	0.9856
	Ao Arch	0.5754	IVC inspiration	0.6466
	Ao Abd	0.8294	IVC expiration	0.8223
Peak O_2_ pulse	Ao Annulus	0.2492	PA	0.8435
	Ao SV	0.9352	LPA	0.7979
	Ao STJ	0.8808	RPA	0.5840
	Ao Arch	0.8399	IVC inspiration	0.7249
	Ao Abd	0.6846	IVC expiration	0.7633
Peak RER	Ao Annulus	0.4723	PA	0.3411
	Ao SV	0.7989	LPA	0.7389
	Ao STJ	0.8924	RPA	0.3881
	Ao Arch	0.8266	IVC inspiration	0.5411
	Ao Abd	0.0738	IVC expiration	0.5185
Peak VCO_2_	Ao Annulus	0.2503	PA	0.7307
	Ao SV	0.7918	LPA	0.9839
	Ao STJ	0.8215	RPA	0.8409
	Ao Arch	0.3871	IVC inspiration	0.9253
	Ao Abd	0.9614	IVC expiration	0.9438
Peak VO_2_	Ao Annulus	0.3614	PA	0.5366
	Ao SV	0.7934	LPA	0.4838
	Ao STJ	0.5722	RPA	0.5008
	Ao Arch	0.9846	IVC inspiration	0.7120
	Ao Abd	0.6848	IVC expiration	0.7032
Peak VO_2_·kg^-1^	Ao Annulus	0.3551	PA	0.7813
	Ao SV	0.7603	LPA	0.9404
	Ao STJ	0.9405	RPA	0.6610
	Ao Arch	0.4909	IVC inspiration	0.8451
	Ao Abd	0.3531	IVC expiration	0.8840
Peak VE	Ao Annulus	0.1126	PA	0.5002
	Ao SV	0.9613	LPA	0.7271
	Ao STJ	0.6552	RPA	0.3120
	Ao Arch	0.6508	IVC inspiration	0.5920
	Ao Abd	0.7369	IVC expiration	0.6840

Multivariate linear regression models analyzed the effects of interactions of vascular dimensions (x) and sex (x × sex) on exercise capacity parameters.

Abbreviations: Ao Abd–abdominal aorta diameter; Ao Annulus–annulus of the aorta diameter; Ao Arch—aortic arch diameter; Ao STJ—aortic sinotubular junction diameter; Ao SV—aortic sinus of Valsalva diameter; HR–heart rate; IVC expiration–inferior vena cava diameter during expiration; IVC inspiration–inferior vena cava diameter during inspiration; LPA–left pulmonary artery diameter; O_2_pulse—the ratio of VO_2_ to HR; PA–main pulmonary artery diameter; RER–respiratory exchange ratio; RPA–right pulmonary artery diameter; SE–standard error; VCO_2_ –the volume of produced CO_2_; VE–minute ventilation; VO_2_ –the volume of consumed O_2_; VO_2_·kg^-1^ –the volume of consumed O_2_ per kilogram of body weight

**Table 4:** Associations between peak CPET parameters with resting dimensions of the great vessels and sex. Data presented for N = 190 (134 males, 56 females).

Dependent y	x	Effects of x			Effects of sex			R^2^ for the model
		Estimate x	SE	p-value	Estimate sex	SE	p-value	
Peak EE	Ao Annulus	0.313	0.096	0.0013	-2.853	0.283	<0.0001	0.555
	Ao SV	0.178	0.068	0.0100	-3.008	0.275	<0.0001	0.544
	Ao STJ	0.092	0.071	0.1926	-3.367	0.252	<0.0001	0.531
	Ao Arch	0.140	0.081	0.0859	-3.230	0.252	<0.0001	0.536
	Ao Abd	0.507	0.115	<0.0001	-2.946	0.246	<0.0001	0.536
	PA	0.268	0.079	0.0009	-3.068	0.248	<0.0001	0.554
	LPA	0.435	0.101	<0.0001	-3.007	0.242	<0.0001	0.569
	RPA	0.361	0.106	0.0008	-3.089	0.245	<0.0001	0.555
	IVC inspiration	0.171	0.058	0.0035	-3.423	0.230	<0.0001	0.548
	IVC expiration	0.142	0.039	0.0003	-3.416	0.227	<0.0001	0.559
Peak HR	Ao Annulus	-0.829	0.363	0.0236	-2.750	1.072	0.0111	0.044
	Ao SV	-1.307	0.243	<0.0001	-4.152	0.974	<0.0001	0.145
	Ao STJ	-1.215	0.250	<0.0001	-2.921	0.893	0.0013	0.122
	Ao Arch	-1.242	0.292	<0.0001	-2.786	0.909	0.0025	0.107
	Ao Abd	-1.625	0.435	0.0002	-2.755	0.933	0.0035	0.107
	PA	-0.993	0.297	0.0010	-2.528	0.929	0.0071	0.068
	LPA	-2.159	0.364	<0.0001	-3.240	0.870	0.0003	0.174
	RPA	-2.130	0.377	<0.0001	-3.126	0.875	0.0004	0.160
	IVC inspiration	-0.312	0.219	0.1571	-1.264	0.876	0.1510	0.023
	IVC expiration	-0.423	0.146	0.0042	-1.254	0.862	0.1472	0.058
Peak load	Ao Annulus	5.803	1.925	0.0029	-46.773	5.681	<0.0001	0.466
	Ao SV	3.773	1.368	0.0064	-48.629	5.491	<0.0001	0.459
	Ao STJ	2.263	1.412	0.1106	-53.826	5.039	<0.0001	0.445
	Ao Arch	3.174	1.620	0.0516	-53.060	5.044	<0.0001	0.450
	Ao Abd	9.254	2.317	<0.0001	-48.617	4.968	<0.0001	0.450
	PA	4.989	1.597	0.0021	-50.723	4.991	<0.0001	0.465
	LPA	7.897	2.043	0.0002	-49.776	4.887	<0.0001	0.479
	RPA	6.638	2.128	0.0021	-51.172	4.938	<0.0001	0.465
	IVC inspiration	3.413	1.154	0.0035	-57.380	4.610	<0.0001	0.463
	IVC expiration	2.800	0.773	0.0004	-57.221	4.556	<0.0001	0.474
Peak O_2_ pulse	Ao Annulus	0.421	0.106	0.0001	-2.609	0.313	<0.0001	0.506
	Ao SV	0.335	0.074	<0.0001	-2.610	0.298	<0.0001	0.514
	Ao STJ	0.241	0.078	0.0022	-3.018	0.277	<0.0001	0.488
	Ao Arch	0.309	0.089	0.0006	-2.971	0.276	<0.0001	0.494
	Ao Abd	0.741	0.124	<0.0001	-2.681	0.265	<0.0001	0.494
	PA	0.389	0.087	<0.0001	-2.862	0.273	<0.0001	0.513
	LPA	0.717	0.107	<0.0001	-2.698	0.255	<0.0001	0.566
	RPA	0.636	0.113	<0.0001	-2.796	0.262	<0.0001	0.540
	IVC inspiration	0.215	0.064	0.0010	-3.372	0.256	<0.0001	0.492
	IVC expiration	0.198	0.042	<0.0001	-3.366	0.250	<0.0001	0.518
Peak RER	Ao Annulus	-0.004	0.002	0.0554	-0.021	0.007	0.0025	0.051
	Ao SV	-0.002	0.002	0.1277	-0.018	0.007	0.0051	0.041
	Ao STJ	-0.004	0.002	0.0081	-0.019	0.006	0.0014	0.065
	Ao Arch	-0.005	0.002	0.0155	-0.018	0.006	0.0019	0.059
	Ao Abd	-0.011	0.003	0.0001	-0.023	0.006	0.0001	0.059
	PA	-0.001	0.002	0.6018	-0.014	0.006	0.0180	0.035
	LPA	-0.008	0.002	0.0006	-0.021	0.006	0.0004	0.090
	RPA	-0.009	0.002	0.0007	-0.020	0.006	0.0005	0.091
	IVC inspiration	0.000	0.001	0.7866	-0.013	0.006	0.0184	0.031
	IVC expiration	-0.001	0.001	0.3095	-0.013	0.005	0.0198	0.037
Peak VCO_2_	Ao Annulus	0.065	0.022	0.0044	-0.706	0.066	<0.0001	0.567
	Ao SV	0.034	0.016	0.0367	-0.745	0.064	<0.0001	0.555
	Ao STJ	0.011	0.016	0.5207	-0.804	0.059	<0.0001	0.545
	Ao Arch	0.020	0.019	0.2910	-0.794	0.059	<0.0001	0.549
	Ao Abd	0.091	0.027	0.0010	-0.737	0.058	<0.0001	0.549
	PA	0.060	0.019	0.0014	-0.744	0.058	<0.0001	0.569
	LPA	0.079	0.024	0.0011	-0.747	0.057	<0.0001	0.569
	RPA	0.063	0.025	0.0130	-0.765	0.058	<0.0001	0.559
	IVC inspiration	0.039	0.013	0.0039	-0.824	0.054	<0.0001	0.564
	IVC expiration	0.030	0.009	0.0010	-0.822	0.053	<0.0001	0.570
Peak VO_2_	Ao Annulus	0.061	0.018	0.0011	-0.528	0.054	<0.0001	0.542
	Ao SV	0.036	0.013	0.0066	-0.555	0.053	<0.0001	0.532
	Ao STJ	0.021	0.014	0.1242	-0.606	0.048	<0.0001	0.519
	Ao Arch	0.031	0.016	0.0443	-0.596	0.048	<0.0001	0.524
	Ao Abd	0.103	0.022	<0.0001	-0.542	0.047	<0.0001	0.524
	PA	0.052	0.015	0.0008	-0.570	0.048	<0.0001	0.541
	LPA	0.090	0.019	<0.0001	-0.553	0.046	<0.0001	0.564
	RPA	0.075	0.020	0.0003	-0.570	0.047	<0.0001	0.546
	IVC inspiration	0.033	0.011	0.0034	-0.639	0.044	<0.0001	0.535
	IVC expiration	0.028	0.007	0.0002	-0.637	0.044	<0.0001	0.548
Peak VO_2_·kg^-1^	Ao Annulus	-0.917	0.431	0.0347	-6.491	1.272	<0.0001	0.139
	Ao SV	0.132	0.309	0.6688	-4.617	1.239	0.0003	0.107
	Ao STJ	-0.574	0.312	0.0673	-5.662	1.112	<0.0001	0.123
	Ao Arch	-0.728	0.358	0.0435	-5.766	1.115	<0.0001	0.126
	Ao Abd	0.730	0.531	0.1715	-4.258	1.139	0.0002	0.126
	PA	0.729	0.358	0.0433	-4.013	1.120	0.0004	0.127
	LPA	1.676	0.453	0.0003	-3.410	1.085	0.0019	0.167
	RPA	1.309	0.473	0.0063	-3.791	1.098	0.0007	0.146
	IVC inspiration	0.651	0.257	0.0121	-5.011	1.026	<0.0001	0.137
	IVC expiration	0.488	0.173	0.0054	-4.974	1.022	<0.0001	0.143
Peak VE	Ao Annulus	2.336	0.913	0.0113	-22.788	2.694	<0.0001	0.460
	Ao SV	1.464	0.648	0.0251	-23.653	2.603	<0.0001	0.454
	Ao STJ	0.032	0.670	0.9623	-26.790	2.390	<0.0001	0.440
	Ao Arch	1.110	0.767	0.1493	-25.517	2.387	<0.0001	0.445
	Ao Abd	2.547	1.122	0.0244	-24.575	2.405	<0.0001	0.445
	PA	1.594	0.763	0.0380	-24.885	2.384	<0.0001	0.453
	LPA	2.210	0.987	0.0263	-24.861	2.361	<0.0001	0.455
	RPA	2.018	1.018	0.0488	-25.115	2.361	<0.0001	0.451
	IVC inspiration	0.814	0.553	0.1427	-26.966	2.209	<0.0001	0.445
	IVC expiration	0.879	0.371	0.0189	-26.958	2.188	<0.0001	0.455

Multivariate linear regression models analyzed the effects of vascular dimensions (x) and sex on exercise capacity parameters

Abbreviations: Ao Abd–abdominal aorta diameter; Ao Annulus–annulus of the aorta diameter; Ao Arch—aortic arch diameter; Ao STJ—aortic sinotubular junction diameter; Ao SV—aortic sinus of Valsalva diameter; HR–heart rate; IVC expiration–inferior vena cava diameter during expiration; IVC inspiration–inferior vena cava diameter during inspiration; LPA–left pulmonary artery diameter; O_2_pulse—the ratio of VO_2_ to HR; PA–main pulmonary artery diameter; R2 –the coefficient of determination; RER–respiratory exchange ratio; RPA–right pulmonary artery diameter; SE–standard error; VCO_2_ –the volume of produced CO_2_; VE–minute ventilation; VO_2_·kg^-1^ –the volume of consumed O_2_; VO_2_kg–the volume of consumed O_2_ per kilogram of body weight;

**Fig 3:**
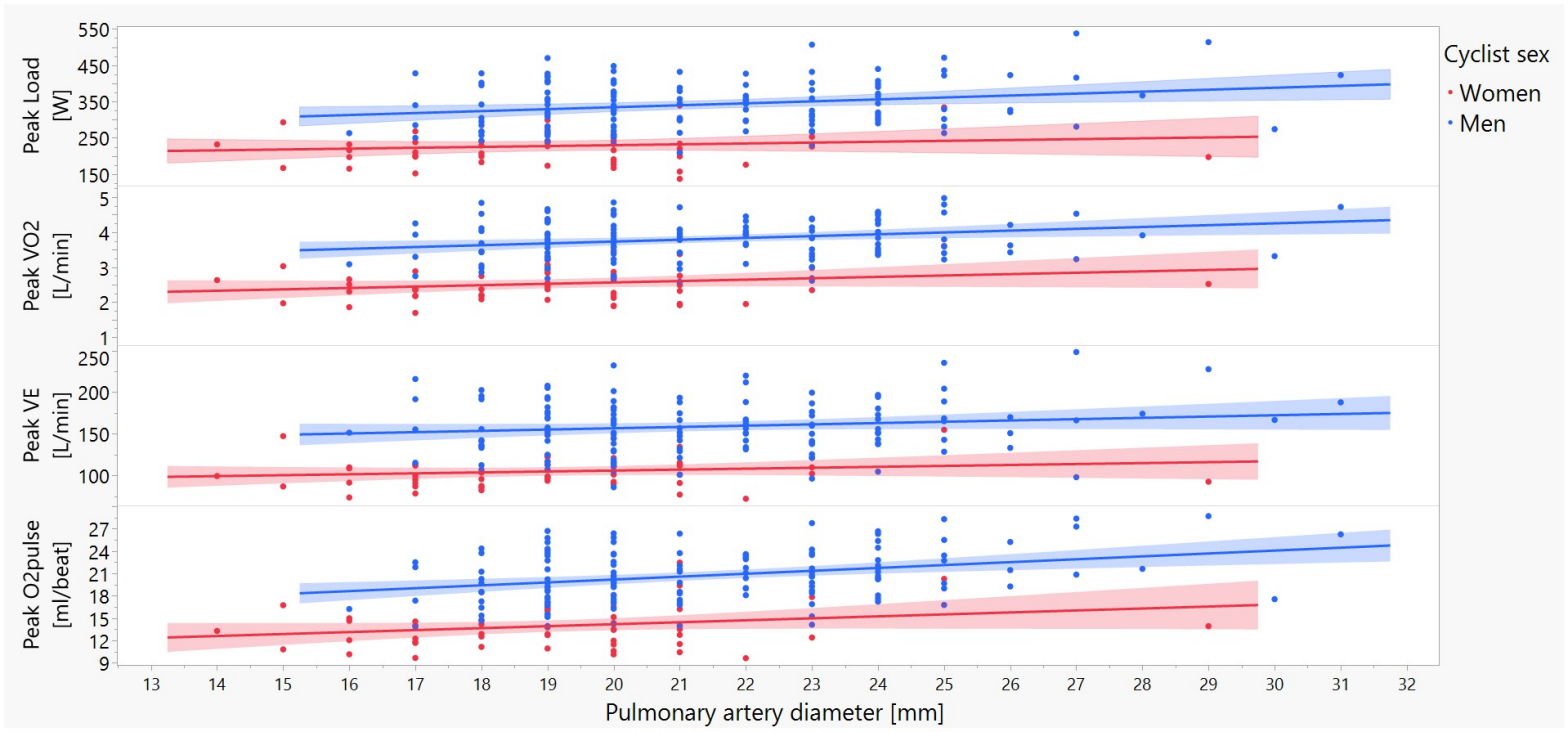
An example of graphic presentation of associations between main pulmonary artery diameter and peak exercise capacity parameters (load, VO_2_, O_2_pulse and VCO_2_). Red dots and shades indicate females, blue dots and shades represents males. Abbreviations: O_2_pulse—the ratio of VO_2_ to HR; VE–minute ventilation; VO_2_ –the volume of consumed O_2_.

Significant associations were found between both peak HR and O_2_ pulse and aortic, PA, and IVC dimensions. In contrast, fewer associations were observed between RER or VE and vascular dimensions. Males and females showed significant differences in the relationships between peak CPET parameters and vessel dimensions. This can be attributed to both higher peak CPET values and larger sizes of great vessel in males than females. Consistently, the correlations between CPET parameters and vessel dimensions were stronger and parallel in males (Fig 3). Notably, the interactions between vessel dimensions and sex did not affect the associations between these variables and CPET results. Taken together, these results suggest that sex differences exist in the baseline values (intercepts) of the relationships between CPET and vessel dimensions but not in the overall trends (slopes) of these associations.

## Discussion

Larger major vessel dimensions, particularly of the aorta and PA, were associated with greater peak exercise capacity in amateur cyclists, regardless of their sex. The strongest correlations were observed between aortic and PA dimensions and peak O_2_ pulse, VO_2_, VCO_2_, VE, and EE. However, these correlations were moderate at best. IVC dimensions showed weak or nonsignificant correlations with peak CPET parameters. When these correlations were analyzed separately for males and females, weaker or nonsignificant associations were found. Linear regression models with interactions showed that male cyclists had larger dimensions of the aorta and PA, and their peak exercise capacity was generally greater than that of females—this was reflected by the intercepts of the regression lines. However, the slopes of the regression lines indicating the strength of the association between CPET parameters and vessel diameter did not differ significantly between the sexes—this was demonstrated by nonsignificant effects of the interactions between vessel dimensions and sex.

### Changes in the vascular system due to exercise

The vascular system transports nutrients and O_2_ to working muscle cells and removes various substances like CO_2_, lactates, excessive H^+^, and heat from them [24–26]. Repeated endurance training involves hours of exercise during which blood flow (including cardiac output and venous return) increases through all arteries and veins to meet the increased metabolic demands of the muscles. Over time, endurance training increases total blood volume by 20–25% in trained individuals and up to 50% in elite athletes. This increase is primarily due to direct increases in red blood cell mass and plasma volume [5].

During exercise, the aorta and arteries must efficiently deliver this increased blood volume to the working muscles. Meanwhile, deoxygenated blood returns from the muscles through the veins, including the IVC, to the right side of the heart, which pumps it through the PA to the lungs for essential gas exchange (O_2_ uptake and CO_2_ removal) and heat dissipation.

Repeated endurance exercise leads to vascular adaptations, which are particularly evident in the microvasculature of trained muscle groups [27]. Muscle capillarization may serve as a limiting factor for exercise performance [28]. Training elevates the number of capillaries per muscle fiber by 10–20% within several weeks in untrained individuals, with a slower rate of increase in well-trained athletes [28]. Not only capillary density, but also capillary positioning may be influential [28]. However, research linking muscle capillarization and exercise performance is lacking [28].

Endurance training also triggers arterial remodeling. Increased circulating blood volume and altered shear stress and transmural pressure lead to a decrease in arterial wall thickness and an increase in lumen diameter [1, 29]. These widened arteries result in reduced vascular resistance, effectively priming the muscles for an increase in blood supply during exercise [1]. The dimensions of the aorta [13, 30–35], PA [36, 37], and IVC [38, 39] are reported to be larger in training athletes than in sedentary controls. Similarly to other studies, we demonstrated that healthy amateur cyclists with greater exercise capacity had larger aortic diameters [13, 30–35]. However, less is known about the PA and IVC and about possible sex differences.

### Arteries of athletes

Repeated exercise improves vascular plasticity and increases the body’s ability to respond to physical activity [29]. Endurance training activates endothelial nitric oxide synthase, leading to the production of nitric oxide, which dilates both arteries and veins [9]. As a result, the large arteries that supply and drain frequently used muscles are enlarged [29, 40]. For example, wheelchair-using athletes have larger aortas, subclavian arteries, and carotid arteries but a smaller IVC and abdominal aortas than controls. In rowers, the brachial artery is disproportionately enlarged [29, 40]. In our study, cyclists with greater exercise capacity had not only a wider thoracic aorta but also a wider abdominal aorta, which supplies blood to the leg muscles used during cycling. This finding was consistent in both male and female cyclists with superior peak CPET results.

### Aortic dimensions of athletes

Athletes have larger diameters of the aortic root than controls [13, 30–35]. However, most of them do not exceed the 99^th^ percentile for males and females (40 and 38 mm, respectively) [30, 31, 33, 35]. Athletes have an aortic diameter that is 3.2 mm larger at the level of the sinuses of Valsalva and an aortic valve annulus that is 1.6 mm larger compared to nonathletic controls [31]. No progression of aortic root enlargement in athletes is observed if the values are less than the 99^th^ percentile [30]. However, if the values are greater and aortic root dilatation is present, it is most probably pathologic and likely to progress [31]. Differences due to athletic discipline and aortic root size have been reported [32, 41]. Athletes who train in sports with a higher dynamic component have a larger aortic root [32]. The diameter of the aortic root is larger in males than in females [31–33]. Similarly, we found that all aortic diameter measurements (annulus, sinus Valsalva, STJ, arch, and abdominal) were greater in males than in females.

### Arterial dimensions and exercise capacity

According to Radegran et al., common femoral artery diameter was correlated with peak VO_2_ during exercise on an ergometer (*r* = 0.91) [42]. Also, Rasica et al. report a strong correlation between resting superficial femoral artery diameter and peak VO_2_, which may represent a key adaptation for active muscles perfusion [27]. Vanhees et al. reported that a 16-week training program (which included 48 h of cycling, jogging, and calisthenics) increased VO_2_; however, it decreased resting brachial artery diameter and mean blood flow velocity in the brachial artery and had no effect on aortic diameter or cardiac output [43]. These results can be explained by the fact that the total training time was short, which could account for the lack of changes in aortic diameter. The decreased brachial artery diameter can be explained by the training program, which heavily favored lower-body workouts (83% of the training time). Another study found that after a 3-month exercise program (indoor cycling 2 or 3 times per week) healthy females had increased diameters of the infrarenal aorta, thoracic aorta, and brachial artery. No differences were reported in aortic root or carotid artery diameters [44]. A positive correlation was found between the absolute change in peak workload and the absolute change in the diameter of the ascending aorta (*r* = 0.42) [44]. Factors associated with aortic size at the sinuses of Valsalva included sex, height, sport type (rowing), and elite competitor status (rowing participation in world championships or Olympics or marathon time under 2 h and 45 min) [34]. We report that increased aortic dimensions in amateur cyclists were associated with better exercise performance during CPET in both males and females.

### Adaptation of the great vessels to exercise and heat redistribution

Heat is a byproduct of metabolism, energy production and energy use in muscles, at rest and during exercise. Heat must be redistributed to other organs, mainly the skin and lungs, to dissipate it from the body to prevent hyperthermia. Increased blood flow through working muscles prevents heat accumulation [45]. Similar physiological responses to exercise are typical of fever. A rise in body temperature during fever is accompanied by skin vessel dilation and increased HR, cardiac output, and ventilation [46]. Repeated exercise does not change the amount of heat produced per exercise bout, but during each bout of exercise the heat accumulation is lower [45]. This is due to increased blood flow through working muscles and an increased release of heat into the blood [45]. The physiology of exercise during heat stress has been studied, but little is known about the mechanisms of vascular adaptation under these conditions or whether greater heat production contributes to long-term vascular remodeling in endurance athletes [4, 5, 47].

In our study, amateur cyclists with larger diameters of the aorta, PA, and IVC had a greater peak exercise capacity and a higher EE. One report suggests that humans have a larger aortic diameter (a surrogate measure for cardiac output), reflecting higher EE, than apes [48]. In a group of patients with abdominal aortic aneurysm, resting EE was higher than in controls [49]. Otherwise, the link between aortic dimensions and EE has not been studied.

Better exercise performance improves heat loss capacity (activation of cutaneous vasodilation and increased blood flow at a lower core temperature, reduction in the internal temperature threshold for the onset of sweating, and increased sweat rate) [4, 5, 50]. One proposed mechanism is that exercise leads to hypervolemia, an increase in total body water volume. This means that a greater volume of interstitial fluid is available for cardiac output and is distributed to working muscles and other organs, including the skin and sweat glands. Increased exercise intensity also increases the total loss of Na^+^ and Cl^−^ ions via sweat [50, 51]. Increased skin perfusion and a greater amount of water available for evaporation through the skin facilitates more efficient heat loss [4, 5, 52]. This training-induced, prolonged hypervolemia may be responsible for the adaptation of great vessels, ultimately leading to improved exercise performance.

### The inferior vena cava during exercise

The maximal expiratory IVC diameter is usually between 15 and 25 mm and is larger in males than in females [38, 53]. However, in patients under 60 years old, sex differences are nonsignificant [38]. Our findings are similar: no differences between males and females in IVC diameter during expiration (20.6 vs. 21.0 mm, respectively) or inspiration (10.2 vs. 10.5 mm, respectively) were found.

Larger expiratory IVC diameters have been reported in trained athletes [38, 39]. Furthermore, IVC diameter correlates with exercise capacity parameters such as VO_2_ [54]. We found correlations between expiratory IVC and peak exercise capacity parameters (HR, VO_2_, O_2_ pulse, and load). This suggests that IVC size can be influenced by endurance training. It is possible that a larger IVC diameter means the individual has a larger reservoir of circulating blood and therefore is better prepared for sustaining longer bouts of exercise. This observation is supported by the clinical measurement of IVC diameter to assess patient volemic status. For example, the IVC diameter is related to percent weight loss after football practice [55]. Patients with hypervolemia have a larger IVC; similarly, athletes with better exercise performance, and therefore a larger volemic reservoir, have a larger IVC.

### Pulmonary arteries during exercise

Athletes have larger PA dimensions than non-athletes, as well as a greater pulmonary vascular reserve, which is essential for accommodating the significantly increased blood flow that occurs during exercise [36, 37]. Athletes with higher pulmonary blood flow tend to have higher peak VO_2_ [36]. Chung et al. reported correlations between dimensions of the PA and aorta (as measured using computed tomography) and exercise capacity (as measured by a 6-min walk test) [56]. We used a more accessible method to measure the dimensions of the PA, namely, transthoracic ECHO. We also used CPET to measure exercise capacity in more detail than provided by the 6-min walk test.

Our results showed that larger dimensions of the main PA, LPA, and RPA were positively correlated with exercise capacity. These associations were observed in both males and females. To date, no studies have specifically analyzed the relationship between PA diameter and exercise capacity using CPET.

### Study limitations

This study was cross-sectional and lacked a control group of sedentary people. During recruitment, we enrolled more males than females (149 vs. 60), mainly due to the consecutive enrollment process. For a various reasons, a higher proportion of adult males are regular cyclists, and our male-female ratio reflects amateur cyclists’ demographics. However, not all echocardiographic measurements were possible for each participant. Thus, complete datasets with the results of CPET and the dimensions of the great vessels were analyzed for 190 subjects (134 males, 56 females). Furthermore, the inclusion criterion for our study was amateur cycling for at least 1 hour per week to include individuals with a large variety of exercise training levels. This allowed us to observe CPET results and associations with echocardiographic parameters in a wide range of exercise levels. However, this resulted in recruiting volunteers with varying exercise levels, who undertook various sports activities. Future studies should include a more homogenous group of highly trained individuals to assess better the impact of exercise training on the dimensions of great vessels. Our results showed more significant associations between the dimensions of great vessels and CPET parameters in males than in females. However, this observation may have been influenced by the unbalanced distribution of the data. If we had included more women, we assume that similar trends would have been found in their exercise capacity. Additionally, it should be noted that although our study’s protocol selection and customization adhered to established guidelines [18, 20], different test protocols can influence peak power achieved during the test. Many studies show minor differences between protocols terminated due to exhaustion, regardless of the test duration or power increments between stages [57–59] Some studies report no differences between test protocols [60]. Finally, it is important to recognize that our study group consisted of volunteers of European descent. Therefore, our results cannot be extrapolated to the general population.

## Conclusion

Males had larger dimensions of the thoracic and proximal abdominal aorta and of the PA and its branches. However, there were no sex differences in the dimensions of the IVC. We observed associations between the dimensions of great vessels and higher exercise capacity parameters (particularly VO_2_, O_2_ pulse, load, and peak EE). These associations were present in both sexes and had comparable directions. The differences in these associations were due to the differing intercepts, which resulted from the observation that males generally have larger vessel dimensions, body size and greater exercise capacity than females. This finding indicates that the direction of changes in the vascular system in exercising males and females is similar and there are no sex-specific changes in this regard. Future studies should determine these findings’ clinical and practical applicability, for example, in health-related screening purposes or tailoring training protocols.

## Supporting information

S1 Table Data used for analyses. (XLSX)
